# Association between *Candida* species and periodontal disease: A systematic review 

**DOI:** 10.18502/CMM.6.2.3420

**Published:** 2020-06

**Authors:** Anjana Suresh Unniachan, Nisha Krishnavilasom Jayakumari, Shruthi Sethuraman

**Affiliations:** 1 Department of Periodontics, Vydehi Institute of Dental Sciences and Research Center, Bangalore, India

**Keywords:** Dental plaque, Periodontal pockets, Yeasts

## Abstract

Periodontal diseases result in the inflammation of the supporting structures of the teeth, thereby leading to attachment loss and bone loss. One of the main etiological factors responsible for this condition is the presence of subgingival biofilms, comprising microorganisms, namely bacteria, viruses, and fungi. *Candida* species is one of the fungi reported to be found in periodontal disease which is suggestive of the presence of an association between these variables. The aim of this systematic review was to evaluate the association of *Candida* species with periodontal disease and determine the prevalence of these species in the patients affected with this disease. The articles related to the subject of interest were searched in several databases, including the PubMed, Web of Science, Google Scholar Medline, Embase, Cochrane Library, and Scopus. The search process was accomplished using three keywords, namely ‘‘*Candida* species’’, ‘‘Chronic periodontitis’’, and ‘‘Gingivitis’’. All the identified studies were comprehensively evaluated for the association of *Candida* species with periodontal disease. This systematic review included 23 articles, which assessed the prevalence of *Candida* species in periodontal diseases. The results of 21 studies were indicative of a positive association between *Candida* species and periodontal diseases. Accordingly, it was concluded that there is a strong association between the presence of *Candida* species and periodontal diseases

## Introduction

he human oral cavity remains to be the most important microbial gateway that harbors a wide variety of environmental microorganisms, mostly bacteria, in addition to fungi, protozoa, and viruses [ 1 ]. This complex microbiome is comprised of an estimated 600 bacterial species [ 1 ] and 100 fungal species [ 2 ]. Among the human fungi species, the members of* Candida* are the most frequently recovered ones. Candidosis or candidiasis caused by *Candida albicans* is considered the most common fungal infection of the human oral cavity. Nonetheless, candidiasis caused by non-*albicans Candida* species, such as *C. tropicalis*, *C. parapsilosis*, *C. krusei*, *C. glabrata*, and *C. dubliniensis*, are also becoming common among certain groups of patients [ 3 ].* Candida* has the ability to form multispecies biofilms; therefore, it is considered a well-known human and animal pathogen that causes polymicrobial diseases [ 4 ].

Periodontitis is one of the earliest human diseases recognized to be associated with mixed-species biofilms. Periodontal disease, or periodontitis, is a chronic inflammatory disease of the periodontium, which comprises of the tissues that surround and support the teeth [ 5 ]. It is caused by a synergistic and dysbiotic dental plaque microbial community, with such keystone pathogens as *Porphyromonas gingivalis*, initiating the disruption of tissue homeostasis [ 6 ]. This dental “plaque biofilm” consists of a multitude of microorganisms, including bacteria, fungi, and possibly viruses [ 7 ]. Several studies have reported increased subgingival colonization by yeasts, particularly C. albicans, in chronic periodontitis patients, compared to that in periodontally healthy subjects [ 8 ]. 

The colonization and proliferation of* Candida* species in the oral mucosa and periodontal pockets are facilitated by the virulence factors they possess. With bacterial species, such as *Porphyromonas gingivalis*, *Tannerella forsythia*, and *Aggregatibacter actinomycetemcomitans* being the most frequently associated periodontal pathogens, the evidence for yeast involvement in periodontal disease remains to be scarce [ 9 ]. The fungi may act directly, in conjunction with subgingival bacterial pathogens, or as a cofactor by inducing the production of pro-inflammatory cytokines, which increase the occurrence of periodontal attachment loss and as a result lead to the occurrence of periodontal disease [ 10 ].

Systematic reviews are designed to assemble, appraise, and make sense of the totality of the evidence available as far as possible [ 11 ]. To the best of our knowledge, no previous systematic review has investigated the role of* Candida* in periodontal disease. Therefore, the aim of this systematic review was to critically and comprehensively evaluate the presence of* Candida* species in periodontal disease.

## Materials and Methods

**Search strategies**

The studies evaluating the presence of* Candida* species in periodontal diseases from September 2003 to January 2019 were searched in several databases, including the PubMed, Web of Science, Google Scholar, Medline, Embase, Cochrane Library, and Scopus. The search was restricted to original articles that were published in English language and addressed the presence of* Candida* species in periodontal disease. The search process was performed considering the following keywords in the medical subject headings, titles, or abstracts of the articles: "Candida species", "Chronic periodontitis", and "Gingivitis". 

The bibliographies of the retrieved articles were also searched for additional references. The titles of the identified articles were examined closely in order to determine the eligibility of the paper to be included in this review. In addition, the references from the selected articles were examined as a further search tool. All papers the keywords of which were present in their titles or abstracts were used in the initial list, and other unrelated articles were eliminated.

**Inclusion criteria**

There was no time restriction for the selection of the articles for the review process. All sources which could give us the relevant data for our systematic review were included in the study. In this regard, all original articles addressing the prevalence or presence of* Candida* species in periodontal diseases were considered. The selection of the articles for review was completed based on three stages, involving the examination of: a) titles, b) abstracts, and c) full texts. 

The articles which fulfilled the STROBE checklist was finally considered for the review. The quality of the studies was assessed according to the variables related to study objectives, study population characteristics, inclusion and exclusion criteria, and data collection method. The validity, explicit findings, and appropriate data analysis methods of the studies were also assessed (low quality<10.5, moderate quality=10.6-16.5, and high quality=16.6-22) [ 12 ].

**Exclusion criteria**

The exclusion criteria included: 1) data insufficiency, 2) non-epidemiological type, 3) investigation of the presence of* Candida* species in diseases other than periodontal diseases, 4) publication in languages other than English, and 5) duplication.

**Data extraction**

The data extracted from the reviewed articles included the name of the first author, year of publication, study location, and sample size, sample age, screening method, specimens, presence of* Candida* species, and virulence factors. The abstract and full-text version of the articles were reviewed independently by two authors. In cases of facing any discordance, the papers were reviewed jointly until the differences were resolved.

## Results

**Figure 1:**
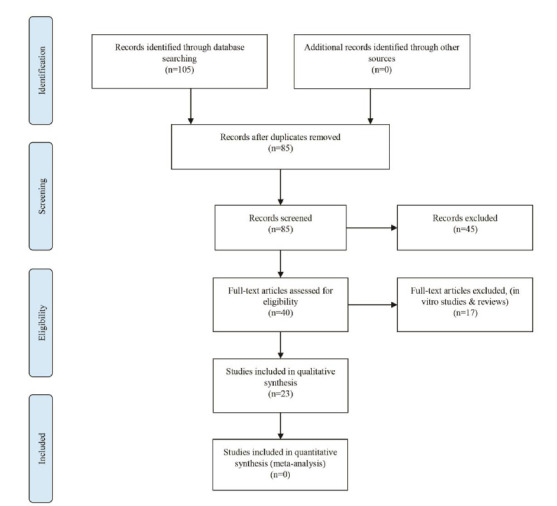
Flow diagram describing the different phases of this systematic review

The search process in the Google Scholar, PubMed, and Scopus databases resulted in the identification of a total of 105 studies. After the removal of duplicate papers, 85 studies remained. In the next stage, 39 cases were excluded following the initial evaluation given the irrelevancy of the titles or topics. Consequently, 46 studies were found to be eligible for the evaluation of the abstract out of which 6 cases were again excluded as the full-text version was not accessible. Then, after the full-text evaluation of the remaining 40 studies, only 23 cases, satisfying the inclusion criteria, were included in the systematic review (Figure 1). The mean score of the STROBE scale obtained for the included studies was 13.8, indicating a moderate quality. The characteristics of the 23 included studies are summarized in Table 1.

**Table 1:** List of studies included in this systematic review

Serial No.	First author	Year	Country	Sample size	Number of groups	Age	Type of disease	Specimen	Methods of identification	Species identified
1	Ja¨rvensivu et al. [24]	2004	Finland	25	1	NS	CP	Subgingival plaque, epithelial, connective tissue sample	PCR	*C. albicans* (16%) *C. albicans* (16%)
2	Urzua et al. [24]	2008	Chile	74	3	20-40 years	CP, AP, PH*	Subgingival plaque, saliva	Biochemical and CHROMagar *Candida*	*C. albicans* (87.5%), *C. dubliniensis* (8.4%), *C. glabrata* (2.6%)
3	Javed et al. [25]	2009	Pakistan	58	1	45-60 years	CP with diabetes	Tongue surface scraping	PCR	*C. albicans* (50%)
4	Alicia et al. [26]	2010	Argentina	82	2	18-70 years	Gingivitis, CP	Subgingival plaque	CHROMagar *Candida*	Gingivitis-*Candida* spp. (57.7%), CP- *Candida* spp. (53.6%)
5	Melton et al. [27]	2010	Mexico	30	2	NS	CP with controlled and uncontrolled diabetes	Saliva, subgingival plaque	CHROMagar *Candida*	CP with controlled diabetes-*C. albicans*(33%),*C. glabrata* (13%),*C. parapsilosis*(0%), *C. tropicalis* (0%)
										CP with uncontrolled diabetes - C.albicans(53%),*C. glabrata* (20%), *C. parapsilosis*(6%), *C. tropicalis* (6%)
6	Sardi et al. [28]	2012	Brazil	11	1	31-68 years	CP with diabetes	Subgingival plaque	PCR	*C. albicans*- 63.3%
7	Mcmanus et al. [29]	2012	Ireland	71	2	21-67 years	CP, PH	Subgingival plaque	CHROMagar *Candida*	CP-*C. albicans* (47.6%), *C. dubliniensis*(23.8%),*C. parapsilosis* (1%), *C. keyfr* (1%), PH-C.albicans (32%), *C. glabrata*(1%)
8	Almubarak et al. [30]	2013	Saudi Arabia	42	1	21-70 years	CP with diabetes	Subgingival plaque	CHROMagar *Candida*	*C. albicans*-38%, *C. dubliniensis*(9.5), C.tropicalis (4.7%), *C. glabrata*(4.7%)
9	Canabarro et al. [8]	2013	Brazil	60	2	31-67 years	CP, PH	Subgingival plaque	Biochemical	CP-30% *Candida*-positive(C.albicans-12, *C. parapsilosis*-2, *C. dubliniensis*-1, C.tropicalis-1) PH-15% *Candida*-positive(C.albicans-3)
10	Joshi PS et al. [17]	2013	India	80	2	40-60 years	CP, PH	Subgingival plaque	Biochemical	*C. albicans*-7.5%
11	Joshi PS et al. [31]	2014	India	80	2	40-60 years	CP,CP with diabetes	Subgingival plaque	Biochemical	CP-*C. albicans*(90%), CP with Diabetes-*C. albicans*(7.5%)
12	Popova et al. [19]	2014	Bulgaria	20	1	NS	CP	Subgingival plaque	PCR	No-*Candida* spp. was isolated
13	Chezian N et al. [32]	2015	India	30	1	35-70 years	CP with diabetes	Subgingival plaque	Sabouraud agar	*C. albicans*-40%
14	Venkatesan et al. [33]	2015	India	30	2	40-60 years	CP , CP with diabetes	Whole saliva	Biochemical tests	CP- *Candida* spp.(13%) CP with diabetes-*Candida* spp.(60%)
15	Arumugam et al. [36]	2015	India	40	2	30.12, 36.71	CP, peri-implatitis	Subgingival plaque	CHROMagar *Candida*	CP-*C. albicans* (14.6%), *C. tropicalis*(2.4%), *C. dubliniensis* (4.9%), C. krusei(4.9%), C.glabrata (0%), *C. parapsilosis*(0%)
										Periimplantitis- *C. albicans* (19.5%), *C. tropicalis* (4.9%), *C. glabrata* (2.4%), *C. parapsilosis*(2.4%), *C. dubliniensis* (0%), C. krusei (0%),
16	Arumugam et al. [36]	2015	India	82	2	19-45 years	CP,CP with patient’s using hormonal contraceptives (HC)	Subgingival plaque	CHROMagar *Candida*	CP with HC-*C. albicans*-54.5%,C.tropicalis-9.1%, C. krusei-18.2%, *C. parapsilosis*-0%,C.dubliniensis-18.2%, *C. glabrata*-0%
										CP-*C. albicans*-66.7%,C.tropicalis-16.7%,C.krusei-0%, *C. parapsilosis*-8.3%,C.dubliniensis-0%, *C. glabrata*-8.3%
17	Babitha et al. [36]	2017	India	30	3	25-50 years	CP,AP,PH	Subgingival plaque	PCR	All the groups in all samples *C. albicans* was isolated
18	Lourenco et al. [22]	2017	Brazil	73	2	NS	Non-HIV infected periodontally healthy, non-HIV infected periodontally affected, HIV-infected periodontally healthy, HIV-infected periodontally affected	Saliva	CHROMagar *Candida*	Non HIV periodontally healthy- C.albicans-58%,*C. tropicalis*-16.7%, C. krusei-0%,*C. parapsilosis*-25%, *C. dubliniensis*-0%,*C. glabrata*-0%
										Non HIV periodontally affected- C.albicans-61%,*C. tropicalis*-16.7%, C. krusei-0%,*C. parapsilosis*-15%, *C. dubliniensis*-0%, *C. glabrata*-0%
										HIV periodontally healthy- C.albicans-58%,*C. tropicalis*-5%, C. krusei-0%, *C. parapsilosis*-5%, *C. dubliniensis*-0%, *C. glabrata*-0%
										HIV periodontally affected- C.albicans-79%,*C. tropicalis*-7%, C. krusei-7%, C.parapsilosis-7%,*C. dubliniensis*-3%, *C. glabrata*-10%
19	Blignaut et al. [23]	2017	Pretoria	87	1	NS	Necrotizing periodontal disease	Tongue swab	CHROMagar *Candida*	*C. albicans*-54%
20	Bhalla et al. [38]	2018	India	45	3	24-65 years	CP, PH, CP with diabetes	Subgingival plaque	Biochemical	PH-0%, CP-Non-*C. albicans*(40%), CPwith DM- Non-*C. albicans*(25%)
21	Lingaiah et al. [39]	2018	India	100	2	NS	CP with type I and CP with type II diabetes	Whole saliva	Sabouraud agar	CP with type 1-*Candida* spp. (48.1%)
										CP with type 2- *Candida* spp. (51.9%)
22	De la Torre et al. [19]	2018	Spain	155	3	30-80 years	CP, PH	Oral rinse, subgingival plaque	CHROMagar *Candida*	CP-OR-*C. albicans* (42%), Non C.albicans(16.1%)
										Plaque- *C. albicans* (28.5%), Non C.albicans (12.5%)
23	Petrovic et al. [40]	2019	Serbia	146	4	NS	PH, CP, CP with controlled and uncontrolled diabetes	Tongue swab, subgingival plaque	CHROMagar *Candida* and biochemical	PH-tongue swab (22.2%), Plaque sample(41.6%)
										CP-tongue swab (16.7%), Plaque sample(26.2%)
										CP with controlled diabetes-tongue swab (21.5%), Plaque sample (53.5%)
										CP with uncontrolled diabetes - tongue swab (47.5%), Plaque sample(72.5%)

*CP: chronic periodontitis, AP: aggressive periodontitis, PH: physically Healthy

Out of 23 studies, 10 and 3 cases had been performed on diabetic and human immunodeficiency virus (HIV)-infected patients, respectively. The remaining 10 studies had been conducted on systemically healthy periodontitis patients. However, out of these studies, on and two manuscripts had also addressed aggressive periodontitis and peri-implantitis, respectively. The age groups of the investigated patients were ≤ 50 and > 50 years in 4 and 12 papers, respectively. However, the age group of the study population was not mentioned in the remaining 7 studies. Regarding sampling, in the majority of the studies (n=18), subgingival plaque was used as the specimen for analysis. However, whole saliva (n=4), oral rinse (n=1), and tongue swab (n=3) were also applied in other studies. Furthermore, with regard to the applied method for organism identification, the majority of the studies used agar or biochemical tests rather than polymerase chain reaction (PCR), which is expensive. Accordingly, PCR was used in only five studies. The results of 22 studies (out of 23 cases) revealed an increased number of* Candida* species in patients with chronic periodontitis, compared to that in healthy subjects, thereby establishing the possibility of the role of this species in periodontal disease. 

## Discussion

Periodontal disease has a polymicrobial etiology. The development of periodontal disease requires the presence of a susceptible host, along with the presence of bacterial plaque. Accordingly, some individuals consider periodontopathogenic bacteria as “required but not sufficient” to cause periodontal disease. However, no disease process results from a single isolated cause or event (i.e., no cause is necessary and sufficient by itself to result in a disease) [ 13 ].

The opportunistic fungus C. albicans has a preponderant role among other species of its genus in periodontal disease. The presence of its hyphae has been demonstrated in the connective tissue of periodontal patients in association with highly invasive anaerobic bacteria, such as *Porphyromonas gingivalis*, *Prevotella intermedia*, and *Aggregatibacter actinomycetemcomitans* [ 14 ].

The exact pathogenic mechanism by which* Candida* species contribute to the progression of periodontal disease is not still known. However, this has been attributed to the known virulent properties of these species, like adhesion, dimorphism, invasion, and biofilm formation. The ability of these species to coaggregate with the periodontal pathogen is also one of the main proposed pathogenic mechanisms. *Candida albicans* colonizes the oral cavity, presenting the commensal or pathogenic properties that can be modified by direct or indirect interactions with different types of bacteria, depending on the localization of the microbial communities (e.g., supragingival plaque, subgingival plaque, and tongue coating). 

*Candida albicans* coaggregates with an obligatory anaerobe *F. nucleatum* [ 15 ] with the engagement of a mannose receptor on the C. albicans surface [ 16 ]. Recent studies have also shown that C. albicans is able to interact with the keystone pathogen of subgingival plaque, *P. gingivalis*, and obligatory anaerobe. However, it is difficult to judge whether the pathogens apply a synergistic or concurrence style of interactions [ 17 ]. The aim of this systematic review was to evaluate the association between the presence of* Candida* species and periodontal diseases. The obtained results revealed a significantly strong positive correlation between the presence of* Candida* species and periodontal diseases.

Joshi et al. stated that C. albicans would have a role in the infrastructure of periodontal microbial plaque, as well as in the adherence of this species to the periodontal tissues [ 18 ]. In a study conducted by De la Toree et al., chronic periodontitis patients were reported to have a higher* Candida* colonization rate than those without chronic periodontitis. However, they were unable to confirm a statistically significant relationship between the colonization of* Candida* and the severity of chronic periodontitis [ 19 ].

In a study carried out by Popova et al. (2014), no* Candida* species was observed in patients with chronic periodontitis. Therefore, among the studies reviewed in the present study, the mentioned study was the only case showing a negative correlation [ 20 ]. Out of the 23 included studies, 10 papers involved the evaluation of the presence of* Candida* species in subgingival microbiota in the patients diagnosed with type I, type II, well-controlled, and poorly controlled diabetes. The results of these studies showed a positive correlation. Diabetic patients have a higher prevalence of C. albicans in the form of subgingival biofilm in the oral cavity, specifically in the periodontal pockets. This could indicate the co-participation of this species in the progression of periodontal disease [ 21 ]. Two of the included studies had been performed on HIV-positive individuals and introduced periodontal disease as a possible factor responsible for the increase in commensal* Candida* species count in HIV-infected patients [ 22 ].

Furthermore, another study included in this review had been performed on HIV-positive individuals with necrotizing periodontal disease. In the mentioned study, tongue swabs were used to investigate the presence of* Candida* species. The results were suggestive of no association between C. albicans and necrotizing periodontal disease. This may be due to the dynamics of the acute inflammatory environment, with an abundance of necrotic, desquamating tissue, associated bleeding, and mediators of acute inflammation, which is not conducive to the undisturbed colonization, replication, and biofilm formation by C. albicans [ 23 ].

During our search for the articles for systematic review, no study was found to assess the effect of periodontal treatment on this opportunistic fungus. The results of all the reviewed studies were suggestive of a positive correlation between the presence of* Candida* species and periodontal diseases; however, the exact pathogenic mechanism is still unknown.

## Conclusion

Based on the results of the reviewed papers, it can be concluded that there is a strong association between the presence of* Candida* species and periodontal diseases. However, further research should be undertaken in this field/area to establish the exact pathogenic mechanism of this opportunistic fungus in periodontal diseases and also confirm the results using a long-term follow-up by evaluating the effect of periodontal treatment on this opportunistic fungus.
